# *Xanthium spinosum* L. Extracts Inhibit Breast Cancer in Mice by Apoptosis Induction and Immune System Modulation

**DOI:** 10.3390/ph15121504

**Published:** 2022-12-02

**Authors:** Lina T. Al Kury, Zainab Taha, Asma Ismail Mahmod, Wamidh H. Talib

**Affiliations:** 1 Department of Health Sciences, College of Natural and Health Sciences, Zayed University, Abu Dhabi 144534, United Arab Emirates; 2 Department of Clinical Pharmacy and Therapeutics, Applied Science Private University, Amman 11931-166, Jordan; 3 Faculty of Allied Medical Sciences, Applied Science Private University, Amman 11931-166, Jordan

**Keywords:** *Xanthium spinosum*, anticancer, immunomodulation, apoptosis, medicinal plant

## Abstract

Plants have been considered for many years as an important source of medicine to treat different diseases. *Xanthium spinosum* L. (Asteraceae, Compositae) is known for its diuretic, anti-inflammatory, and sedative effects. It is also used in the treatment of several ailments, such as cancer. In order to evaluate the anticancer and immunomodulatory activities, crude ethanol extract was prepared from the aerial part of *X. spinosum* and then fractionated using solvents with different polarities. As well, the chemical composition of *X. spinosum* extract and fractions were identified using LC-MS analysis. The antitumor effect of *X. spinosum* was assessed in both in vitro and in vivo models. Apoptosis induction was measured in vitro using a caspase-3 activity kit. Lymphocyte proliferation and phagocytosis and pinocytosis induction were used to quantify the effect of the plant extract and fractions on acquired and innate immunity, respectively. The effect of *X. spinosum* extract, and fractions on the levels of cytokines (IFN-γ, IL-2, IL-4, and IL-10) in murine lymphocytes was determined using a mouse-uncoated TH1/TH2 ELISA kit. Results showed that ethanol extract had the highest antiproliferative activity (IC₅₀ = 2.5 mg mL^−1^) against EMT6/P cell lines, while the aqueous and chloroform fractions had the highest apoptotic activity with 2.2 and 1.7 folds, respectively. On the other hand, the *n*-hexane fraction was the most effective in stimulating lymphocyte proliferation, whereas ethanol extract, aq. Methanol and aqueous fractions exhibited the highest phagocytic activity. As well, *X. spinosum* extract and fractions were able to modulate the expression of IL-2, IL-4, and IFN-γ. A remarkable decrease in tumor size was accomplished following the treatment of tumor-bearing mice with *X. spinosum* extract and fractions. Both aq. Methanol and chloroform fractions showed the highest percentage change in tumor size with -58 and -55%, respectively. As well, tumor-bearing mice treated with chloroform fraction demonstrated a high curable percentage with a value of 57.1%. Anyway, *X. spinosum* extract and fractions exhibited no toxic impact on the liver or kidney functions of the mice-treated groups. These findings may confirm that *X. spinosum* has favorable anticancer and immunomodulatory effects. However, additional studies are required to fully understand the mechanisms of action of this plant and the signaling pathways involved in its effects. Moreover, more testing is needed to have better insight into the apoptotic pathway and to know the exact concentration of active compounds.

## 1. Introduction

Cancer is among the main causes of death, responsible for one in eight deaths worldwide [1]. Cancer deaths are estimated to reach up to 11.5 million in 2030 [2]. Chemotherapy, one of the most common treatments routinely used for cancer, is not devoid of side effects and intrinsic problems [3]. Furthermore, many therapeutic agents have little impact on survival rates. Other treatment options for cancer include surgery and radiation therapy which also have limitations and side effects [4]. Regardless of the successes in treating and managing cancer, the need for more effective anticancer agents that have fewer side effects remains crucial.

Significant scientific evidence shows the importance of medicinal plants in the development of new drugs to treat different diseases. A total of 35,000 plant species have been screened by the National Cancer Institute (NCI) for their anticancer potential. Approximately, 25% of all prescriptions contain a minimum of one active ingredient obtained from medicinal plants [5]. The species *X. spinosum* L. (Asteraceae, Compositae) is a member of the subtribe Ambrosiinae (Heliantheae) [6]. The plant is indigenous to South America, but it is widely distributed in different parts of the world, including the Mediterranean region [7]. *X. spinosum* L. is a source of many flavonoids, including quercetin, iocein, centaurin, pendulin, and patuletin 3-O-glucoside [8]. In addition, several xanthanolide sesquiterpene lactones, such as xanthatin, xanthinin, stizolicin, and solstitialin are found in this plant [9]. *X. spinosum* has been recognized in traditional medicine for its antibacterial [10], antiviral [11], anti-inflammatory [12], and sedative [13] properties. Different plant parts have been used to cure cancer, diarrhea [8,14], rabies [15], and rheumatoid arthritis [16]. Despite the scarcity of available data on the biological and pharmacological effects of *X. spinosum*, the chemical analysis identified a high number of monoterpenes and sesquiterpenes, many of which are known for their antioxidant and anticancer properties [17]. In light of the traditional use of the plant, the current work aimed to explore the anticancer and immunomodulatory activities of *X. spinosum.*

## 2. Results

### 2.1. LC-MS Analysis of X. Spinosum Ethanol Extract

The result of LC-MS analysis showed that ethanol extract, chloroform, and *n*-hexane fractions were rich in eupatilin, with a relative percentage among detected compounds of 89.8%, 76.8%, and 85.5%, respectively (Table 1, Table 2 and Table 3). On the other hand, aqueous and aq. Methanol fractions revealed the presence of apiin at percentage values of 39% and 31%, respectively (Table 4 and Table 5). Acteoside (48.3%) was found abundantly in aq. Methanol fraction, while genistein (19.8%) and kaempferide (19%) were prominently found in aqueous fraction (Table 4 and Table 5).

### 2.2. Antiproliferative Activity of X. spinosum Extract and Fractions

**Figure 1:**
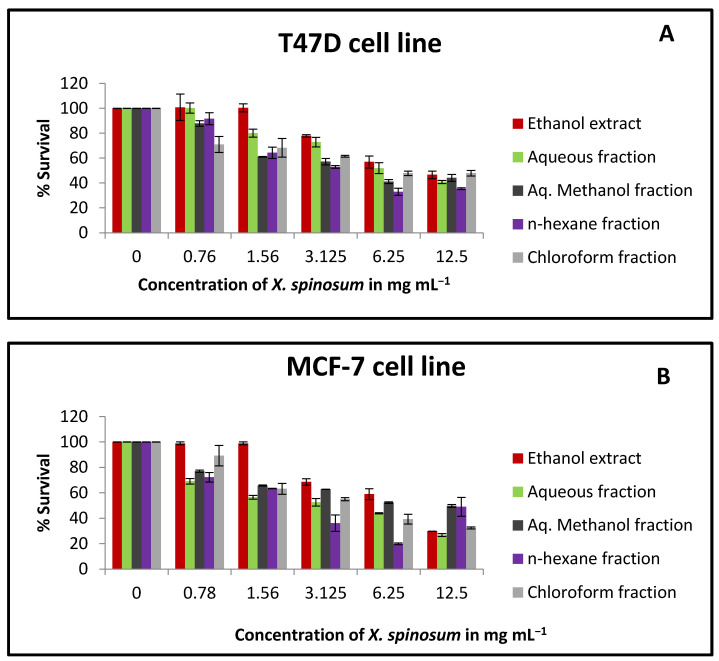

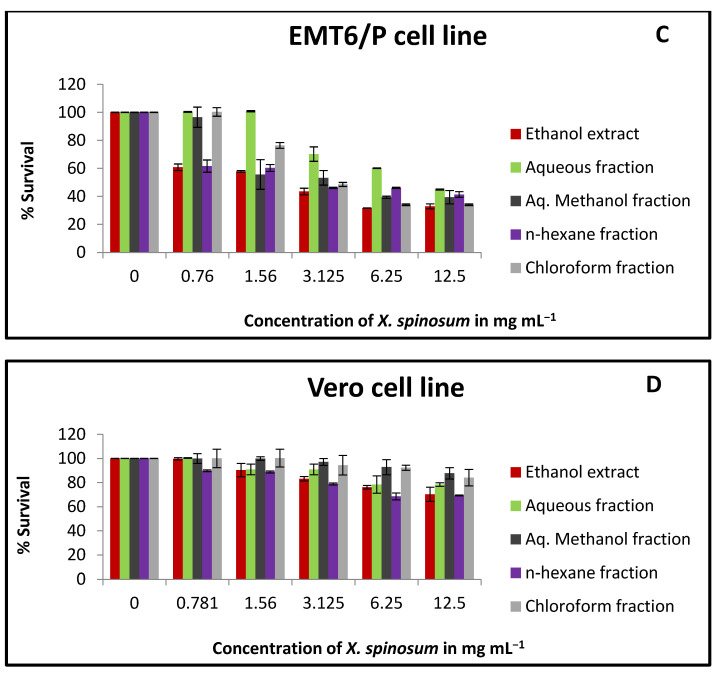
The antiproliferative activity of *X. spinosum* extract and its fractions against multiple cell lines (**A**) T47D breast cancer cells; (**B**) MCF-7 breasts cancer cells; (**C**) EMT6/P mouse breast cancer cells and (**D**) Vero normal cells. The following concentrations were used 0.78 to 12.5 mg mL^−1^. The percentage survival (%) was obtained as (OD of treated cells/OD of control cells ∗ 100). Results are expressed as means of three independent tests (bars) ± SEM (lines).

Based on MTT results, *X. spinosum* extract and fractions exhibited cytotoxic activity against T47D, MCF-7, and EMT6/P cell lines (Figure 1). The ethanol extract was more cytotoxic against EMT6/P compared to T47D and MCF-7 cell lines with an IC₅₀ of 2.57 mg mL^−1^ (Table 6). In the MCF-7 cell line, aqueous and *n*-hexane fractions were able to reduce cell growth effectively with IC₅₀ values of 4.78 and 4.29 mg mL^−1^, respectively. *X. spinosum* extract and fractions showed a higher percentage of survival in normal cells (Vero cells) compared to the tumor cells (Figure 1D).

### 2.3. Apoptotic Activity of X. spinosum Extract and Fractions against EMT6/P Cell Line

**Figure 2:**
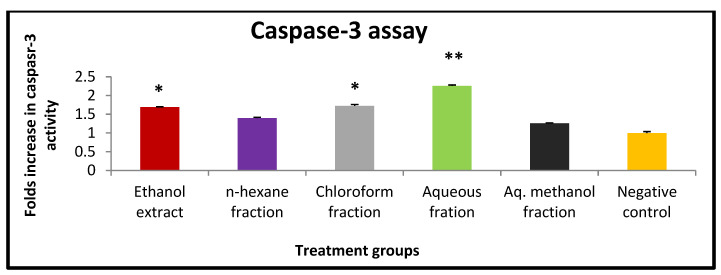
The effect of IC₅₀ concentration of *X. spinosum* extract and fraction on caspase -3 expression in EMT6/P cell line. The concentration of the extract and fractions: ethanol (2.5 mg mL^−1^), chloroform (7.5 mg mL^−1^), aqueous (11 mg mL^−1^), aqueous methanol (8.1 mg mL^−1^), and *n*-hexane (5.5 mg mL^−1^). Results are expressed as means of three independent experiments (bars)± SEM (lines). (** *p* <0.001, * *p* < 0.01 compared to the control).

Apoptosis is controlled by many factors, and one of the main players in this process is caspase-3 protein. In order to detect this apoptotic marker, a caspase-3 assay kit was utilized. Based on the results, the aqueous fraction significantly (*p* < 0.001) showed the highest fold enhancement in caspase-3 activity (2.25 folds), followed by the chloroform fraction (1.72 folds) (Figure 2). However, aq. Methanol fraction showed the lowest effect on the activity of caspase-3 (1.25 folds) compared to the negative control.

### 2.4. Toxicity Evaluation of X. spinosum Extract and Fractions

The LD₅₀ was detected based on the arithmetical method of Karber. There was diversity in the mortality of mice within the three groups (Table 7 and Table 8). The estimated LD₅₀ of *X. spinosum* extract and fractions were as the following: ethanol (1959 mg kg^−1^), aqueous, and aq. Methanol fraction (2709 mg kg^−1^), chloroform (219 mg kg^−1^), and *n*-hexane (167 mg kg^−1^) (Table 7 and Table 8). It is worth mentioning that choosing starting doses were based on a pilot study conducted previously, which revealed a notable safety of the polar extract and fractions. As a result, the preliminary therapeutic dose for the in vivo treatment was 1/10 of the LD₅₀ value for *X. spinosum* extract and fractions.

### 2.5. Antitumor Effects of X. spinosum Extract and Fractions on EMT6/P Cells Implanted in Mice

**Figure 3:**
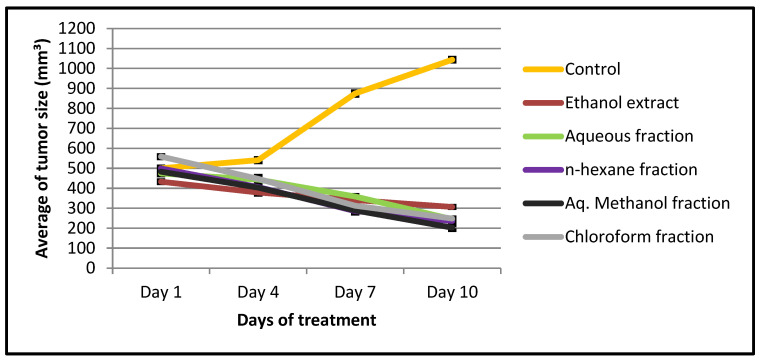
A plot demonstrated the changes in average tumor size (mm^3^) vs. time (days) of treatment with *X. spinosum* extract and fractions in mice inoculated with the EMT6/P cell line. Tumor size is significant (*p* < 0.05) compared to the negative control.

**Figure 4:**
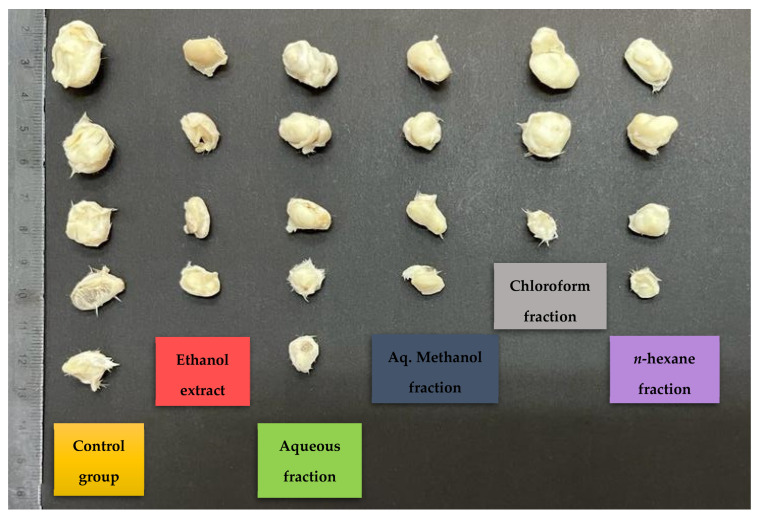
Effects of *X. spinosum* extract and fractions on tumor size and cure percentage. Treatment with *X. spinosum* resulted in a smaller tumor size and higher cure percentage compared to the negative control (*n* = 7 mice in each group).

The in vivo experiment showed a significant (*p* < 0.05) decrease in tumor size (percentage between -58 and -29) compared to the negative control (108.5) (Table 9 and Figure 3). The chloroform fraction-treated group showed 57.1% of mice with no detectable tumor, while the percentage in the control group was 22.2% (Table 9). In addition, there was a difference in the average tumor weight of both groups (Table 9 and Figure 4). Based on the daily observation, mice showed normal activity with no side effects upon treating them with *X. spinosum* extract and fractions.

### 2.6. Effect of X. spinosum Extract and Fractions on Serum Levels of ALT, AST, and Creatinine

**Figure 5:**
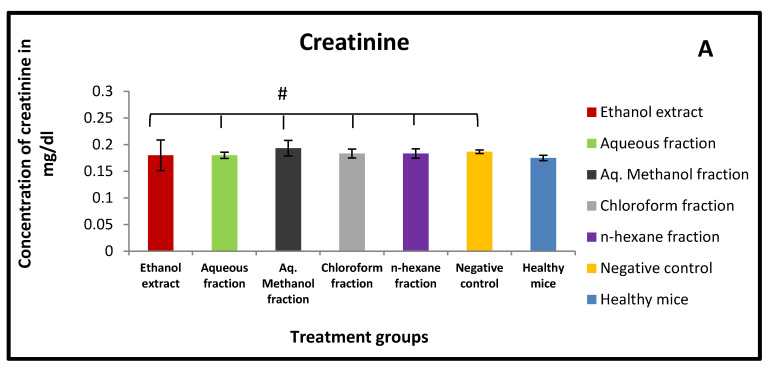

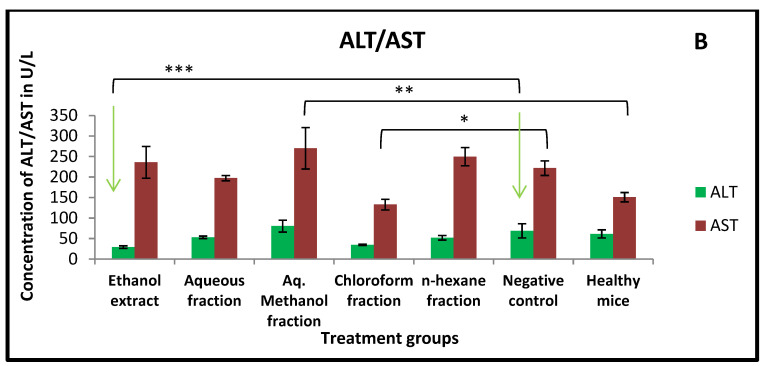
Effects of *X. spinosum* extract and fractions on (**A**) Creatinine (**B**) ALT and AST serum level after 10 days of treatment. (# *p* > 0.05) (* *p* = 0.004) (** *p* = 0.006) (*** *p* = 0.03).

Treating mice with *X. spinosum* extract and fractions exhibited no effect on the serum level of creatinine (Figure 5A). As well it showed an insignificant (*p* > 0.05) change in ALT and AST serum levels compared to the control group (Figure 5B).

### 2.7. Effect of X. spinosum Extract and Fractions on Lymphocytes Proliferation in the Presence and Absence of Mitogens

**Figure 6:**
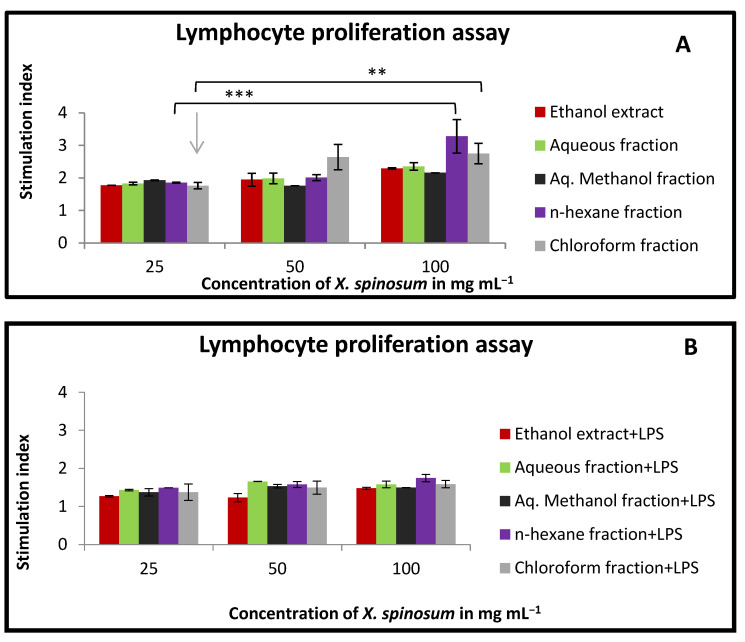

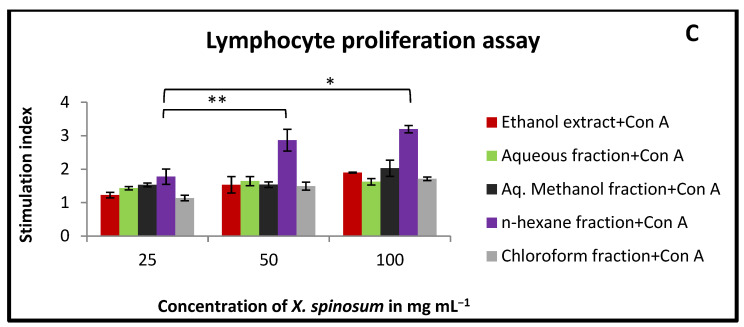
The effect of *X. spinosum* extract and its fractions on the proliferation of lymphocytes extracted from mouse spleen at the concentration range of (25–100 mg mL^−1^). (**A**) In the absence of mitogens); (**B**) with (4 μg mL^−1^) of LPS; (**C**) with (5 μg mL^−1^) of Con A. Results are summarized as means (bars) ± SEM (lines). Stimulation index = stimulated cells (treated)/non-stimulated cells (control). (* *p* = 0.005) (** *p* = 0.01) (*** *p* = 0.004). The results of the other treated groups were insignificant (*p* > 0.05) compared to the control.

Lymphocyte proliferation was slightly stimulated when treated with *X. spinosum* extract and fractions. As a matter of effectiveness, the *n*-hexane fraction showed the highest stimulation index in both conditions, with or without co-mitogen (stimulation index between 3.27 and 1.74) (Figure 6). At the higher tested concentration (100 mg mL^−1^), the chloroform fraction exhibited activity parallel to the *n*-hexane fraction with a stimulation index range between 2.7 and 1.58 (Figure 6).

### 2.8. Effect of X. spinosum Extract and Fractions on Phagocytic Activity of Mouse Peritoneal Macrophages

**Figure 7:**
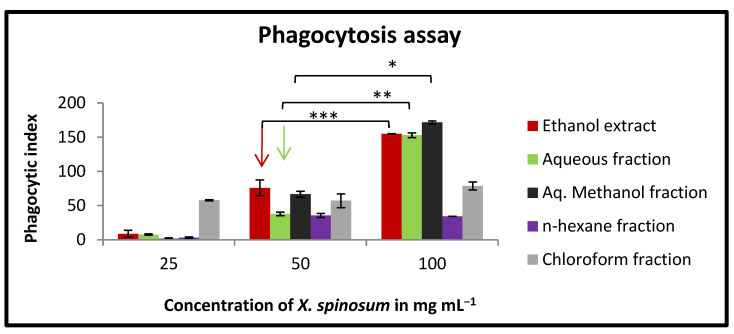
In vitro phagocytic assay using nitro blue tetrazolium (NBT) reduction test of peritoneal macrophages treated with various concentrations (25–100 mg mL^−1^) of *X. spinosum* extract and fractions. Aqueous methanol fraction had the highest phagocytic index (171). Results are expressed as means (bars) ± SEM (lines). Phagocytic index = (OD sample − OD control)/OD control × 100. (* *p* = 0.002) (** *p* = 0.007) (*** *p* = 0.004).

The results of this experiment have shown an improvement in the phagocytic effect of murine macrophages. At concentration of 100 mg mL^−1^, aq. methanol fraction, followed by ethanol extract and aqueous fraction, exhibited the highest activity with a phagocytic index between 171 and 152 (Figure 7). However, the *n*-hexane fraction showed a lower phagocytic index compared to the other *X. spinosum*-tested samples.

### 2.9. Effect of X. spinosum Extract and Fractions on the Pinocytic Activity of Mouse Peritoneal Macrophages

**Figure 8:**
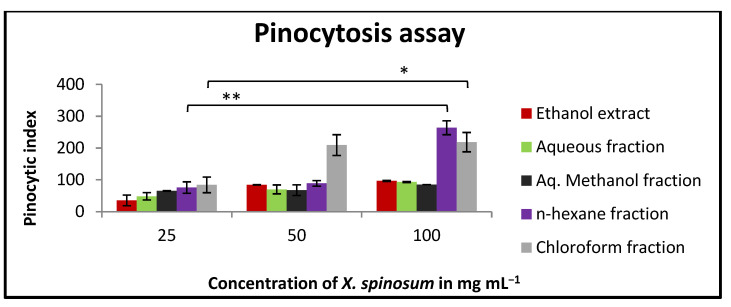
In vitro pinocytic assay using the neutral red method on peritoneal macrophages treated with different concentrations (25–100 mg mL^−1^) of *X. spinosum* extract and fractions. *N-hexane* fraction showed the highest pinocytic index (263). Results are expressed as means of three independent experiments (bars) ± SEM (lines). Phagocytic index = (OD sample − OD control)/OD control × 100. (* *p* = 0.005) (** *p* = 0.003).

Both *n*-hexane and chloroform fractions displayed a slight stimulation of murine macrophage pinocytic activity. At the higher tested concentrations, their pinocytic index values were 263 and 218, respectively (Figure 8).

### 2.10. Effect of X. spinosum Extract and Fractions on Cytokines of Murine Splenic Lymphocytes

**Figure 9:**
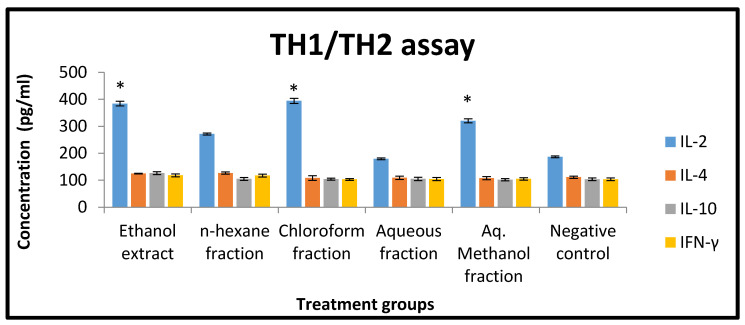
The effect of *X. spinosum* extract and fractions (at a concentration of 1 mg mL^−1^) on the expression level of different cytokines (pg mL^−1^). Results are expressed as means of three independent experiments (bars) ± SEM (lines). (* *p* < 0.05)). Treated groups are compared to the negative control.

The concentration of cytokines (IFN-γ, IL-2, IL-4, and IL-10) was determined using a mouse-uncoated TH1/TH2 ELISA kit. Based on the results, *X. spinosum* extract and fractions were significantly (*p* < 0.05) able to modulate interleukins expression compared to the negative control (Figure 9). Chloroform fraction and ethanol extract showed an effect on IL-2 with a concentration of 394 and 384 pg mL^−1^ compared to the negative control (186 pg mL^−1^) (Figure 9).

## 3. Discussion

Cancer is a serious global health issue and the main cause of mortality responsible for millions of deaths all over the world every year. It is expected that cancer cases will reach up to 24 million in 2030 [18]. Despite the progress in cancer research and therapeutic advancements over the past decade, there remains a need to further investigate potential anti-cancer agents.

Natural products have been long considered an origin of chemical diversity, providing the ground for the identification of promising new anticancer agents that may exhibit high efficiency and low toxicity. *X. spinosum* has been utilized in traditional medicine as a remedy for many different conditions. For example, the leaves and fruits were used for their diuretic and sedative effects, while the infusion of the root was reported to be an emetic [13]. The plant has also been used for its antibacterial, antifungal, and wound-healing effects [19].

This study evaluated the anticancer and immunomodulatory potential of *X. spinosum* extracts. Results showed that *X. spinosum* could upregulate the immune system and consequently suppress cancer growth. Based on the results of the antiproliferative study, *X. spinosum* extract and fractions showed a different magnitude of cytotoxicity against different cancer cell lines, with a high rate of survival in normal cells. Ethanol extract reported the highest effect and suppressed EMT6/P cells at a concentration of 2.57 mg mL^−1^. Aqueous and Aq. Methanol extracts were also able to reduce cell growth effectively. In agreement with these results, an earlier study has shown that methanol extracts of *X. strumarium* possess anticancer activity against different cell lines with IC_50_ values comparable to the values observed in the current study [20,21]. In addition, Romero et al. 2015 reported that xanthatin extraction from *X. spinosum* has potent cytotoxic and anti-angiogenesis effects [8].

To further understand the mechanism by which *X. spinosum* extracts exert their effects, the induction of apoptosis was assessed using a caspase-3 activity assay. Apoptosis inhibition is known to be one of the means by which cancer cells assure proliferation and survival. Therefore, apoptosis induction was proposed as an efficient mechanism to oppose cancer cell proliferation. *X. spinosum* extract and fractions were examined at IC_50_ values that represent their apoptotic effect, and the results revealed that the aqueous fraction had the highest impact. Caspase-3 activity was enhanced by 2.25 folds compared to the negative control. Other solvent extracts exhibited mild to low apoptotic effects.

The pronounced apoptosis induction could be justified by the high percentage (89.82%) of eupatilin present in ethanol extract. This percentage was calculated based on detected compounds in the extract and cannot be used as an exact value. It showed the availability of Eupatilin at high levels among the detected compounds. Eupatilin is a pharmacologically active flavone that was reported to inhibit cell proliferation and migration and promote apoptosis in earlier studies [22,23]. For example, eupatilin was found to suppress the proliferation of malignant human endometrial cells via the upregulation of the expression of P21 and the induction of G2/M cell cycle arrest [24]. In esophageal cancer, eupatilin was found to prevent proliferation via the inhibition of AKT and mitogen-activated protein kinase (MAPK) signaling pathways [25]. Similarly, eupatilin was found to promote reactive oxygen species production and suppress the AKT signaling pathway in renal cell carcinoma [26]. Furthermore, eupatilin was able to reduce cell proliferation and migration in prostate cancer by increasing the mRNA expression of p53, p21, and p27, which induced cell cycle arrest. As well it has prevented cell migration through the down-regulation of PTEN and NF-KB signaling [27].

The cells and organs that comprise the human immune system function in a complex sequence to provide protection against various pathogens. In our study, we assessed the alteration in both the innate and adaptive immune responses after treating the cells with the extracts from *X. spinosum*. In the lymphocyte proliferation assay, the *n*-hexane fraction exhibited the most pronounced stimulation index in the presence and absence of mitogens (LPS and Con-A). Ethanol extract, aq. Methanol fraction and aqueous fraction were effective in stimulating the innate immune response at a concentration of 100 mg mL^−1^. Such an effect was through the enhancement of macrophage phagocytic activity. The highest enhancing effect was reported for the aq. Methanol extract. It seems that the compound eupatilin identified in a relatively high percentage in the ethanolic extract contributes to the immunomodulatory effect by regulating the function of lymphocytes. Eupatilin was reported to reduce the expression of IL-4, TNF-α, IFN-γ, and IL-1β in a mouse model study [28]. Eupatilin was reported to reduce the expression of IL-4, TNF-α, IFN-γ, and IL-1β in mice model study [28]. Moreover, acteoside showed an immunomodulatory effect. It has significantly reduced TNF- α, IFN- γ, and IL-10 in an animal model study of acute colitis [29].

Since *X. spinosum* extract and fractions exhibited a significant antiproliferative effect, there was a further investigation in a mouse model implanted with breast cancer. This revealed a reduction in tumor size accompanied by an increased cure percentage. This regression in tumor size might be attributed not only to the presence of eupatilin in ethanol extract but also to other anticancer compounds such as the flavonoids kumatakenin [30] and kaempferide [31] that may exert their therapeutic potential synergistically to suppress cancer progression by enhancing the immune system and promoting apoptosis. Interestingly, recent findings have shown that kumatakenin was able to induce apoptosis of ovarian cancer cells and inhibit the alternative stimulation of macrophages associated with tumors [32]. Kaempferide was also shown to induce apoptosis in cervical cancer cells and activate the caspase cascade [33].

Romero et al. (2015) identified the sesquiterpene lactone xanthatin in the aqueous extract of *X. spinosum* [8]. The compound has exhibited a noticeable antitumor effect against different cancer cells. For example, xanthatin was reported to inhibit proliferation and promote apoptosis in human breast cancer cells, non-small cell lung cancer cells, and human gastric carcinoma cells [32,33,34]. Such effects agree with the observed activity of the aqueous extract of *X. spinosum* in this study. The aqueous extract was able to reduce cell growth effectively with an IC_50_ value of 4.78. Furthermore, it showed the highest fold increase in caspase-3 activity compared to other extracts and fractions. Therefore, it can be concluded that some of the observed effects are attributed to the presence of xanthatin in the aqueous extract.

## 4. Materials and Methods

### 4.1. Plant Collection and Extract and Fractions Preparation

*X. spinosum* was provided by the United Arab Emirates retail. The plant was classified and authenticated by a plant expert in the Royal Society for the Conservation of Nature, Amman, Jordan. After drying the plant at a suitable temperature, it has ground and converted to a powder to ease extract preparation. The plant powder was soaked in 70% ethanol solvent (Laboratory Chemicals, Bernd Kraft GmbH, Duisburg, Germany) in a proportion of one liter per 100 g of the dried powder. Following three days of maceration accompanied by daily stirring, the maceration suspension was filtered and dried utilizing a rotary evaporator. The drying process was enhanced by using a lyophilizer, and then the concentrated ethanol extract was stored at −20 ℃ till used. Ethanol extract fractionation was the next step by using different solvents with various polarity indexes to condense the main phytochemicals based on their polarities. Liquid-liquid fractionation of the ethanol extract was performed using water, aqueous methanol, chloroform, and *n*-hexane solvents (Laboratory Chemicals, Bernd Kraft GmbH, Germany). The process involved mixing 100 g of ethanol extract with 100 mL of two solvents: chloroform and water and leaving them overnight in a separating funnel. A rotary evaporator was used to evaporate the collected solvents. Afterward, the fractionation method was applied to chloroform fraction using *n*-hexane and aqueous methanol following the same previous steps. The outcomes of the extraction are ethanol crude extract, along with four fractions (aqueous, chloroform, *n*-hexane, and aqueous methanol) [35].

### 4.2. LC-MS Measurements of X. spinosum Ethanol Extract and Fractions

To make the samples ready to use, they dissolved in 2 mL of dimethyl sulfoxide (Scharlab, Barcelona, Spain) and completed the volume to fifty milliliters with acetonitrile (Scharlab, Barcelona, Spain). Centrifugation of the tested samples was carried out at (4000 rpm for 2 min. Then one milliliter was moved to the auto-sampler. The volume that was used to inject the sample was three micro-milliliters. The experiment was achieved utilizing Burker Daltonik (Berman, Germany) impact Ⅱ ESI-Q-TOF system provided with the Burker Dalotonik Elute UPLC system (Beremen, Germany). The apparatus was run using the Ion Source Apollo Ⅱ ion funnel electrospray source (capillary voltage, 2500 v; nebulizer gas, 2 bar; dry gas flow, 8 L min^−1^; dry temperature, 200 ℃; mass accuracy, less than 1 ppm; mass resolution, 50,000 FRS; the TOF repetition rate, 20 kHz). Chromatographic separation was done using a Burker solo 2-C-18 UHPLC column (100 mm × 2.1 mm × 2 μm) at a flow rate of 0.51 mL min^−1^ and a column temperature of 40 ℃. The standard of the analysis was applied to detect two parameters: the ms/z and the retention time.

### 4.3. Animals

Experiments were established using Balb/C female mice with ages around four to six weeks and weights between 21 to 25 g. The animals were kept under specific conditions, including humidity of less than 60% and suitable temperature (25 ℃) with ongoing air ventilation. The Research and Ethical Committee of Applied Science Private University has approved all the murine experiments (Approval Number: 2015-PHA-05). Seventy-six mice were used in this research.

### 4.4. Cell lines and Cell Culture Conditions

In order to investigate the anti-proliferation activity of *X. spinosum* extract and fractions, T47D and MCF-7 (human epithelial breast cancer cells) were used, along with EMT6/P (mouse mammary cells) and Vero (kidney epithelial normal cells from African green monkey). The European Collection of Authenticated Cell Cultures (ECACC, Salisbury, UK) is the source of all the cell lines. The normal cell (Vero) was utilized to evaluate the toxicity of *X. spinosum* extract and fractions in vitro. EMT6/P cells were inoculated into the animals to induct breast tumors in mice. The tested cells were growing in a complete medium and incubated under specific conditions (5% CO₂ and 95% humidity). RPMI 1640 medium (PAN-biotech, Aidenbach, Germany) was used to culture the human breast cancer cells (T47D and MCF-7), while MEM medium (PAN-biotech, Aidenbach, Germany) was used to culture murine breast cancer cells (EMT6/P). For culturing normal cells (Vero), a DMEM medium was applied. The media was supplemented with 1% L-glutamine (Sigma, St. Louis, MO, USA), 10% fetal bovine serum (Gibco, UK), 1% penicillin-streptomycin (Sigma, St. Louis, MO, USA), and 0.1% gentamycin solution (Sigma, St. Louis, MO, USA).

### 4.5. Anti-Proliferation Assay

The cell viability was detected utilizing a 3-(4, 5-dimethylthiazol-2-yl)-2, 5-diphenyl tetrazolium (MMT) (Sigma, St. Louis, MO, USA) assay [36]. The tested cells were seeded (15,000 cells/well) into a flat-bottomed 96-well plate. Following 24 h of incubation, *X. spinosum* was added to the wells at different concentrations (12.5–0.78 mg mL^−1^), applying the serial dilution method. The treated cells were incubated for 48 h. Afterward, the old media was discarded and replaced with fresh media, along with adding 10 µL of MTT solution to each well and incubated for 3 h at 37 ℃. The DMSO was used to dissolve the formazan crystals by adding 100 µL/well and incubating for another 1 h. The absorbance was measured at 550 nm using a microplate reader. The results were represented by calculating the percentage of survival and IC₅₀ values (the half-maximal inhibitory concentration). The negative control in this experiment is the untreated cells. SPSS was used to estimate the concentration of the IC₅₀ (Equation (1)).

Percentage of Cell Viability (%) = (OD of treated cell/OD of control cell) × 100(1)

### 4.6. Determination of Apoptosis in EMT6/P Cells

Evaluating caspase-3 expression can reflect the activity of apoptosis within cancer cells. To start with the protocol of the assay kit, cancer cells (EMT6/P) were cultured in 25 cm^2^ tissue culture flasks with a density of 15,000 cells/mL and incubated for 24 h. Based on the values of the IC₅₀, the concentrations of the *X. spinosum* extract and fractions were determined. After treating the cells with *X. spinosum* (ethanol 4.5 mg mL^−1^, chloroform 9.5 mg/mL, aqueous 13 mg mL^−1^, aqueous methanol mg mL^−1^, and *n*-hexane 10 mg mL^−1^), the treated cells incubated for 48 h. Afterward, the remaining attached cells were collected and centrifuged, and the cell pellet went under many steps according to the kit instructions (caspase-3 assay kit, Abcam, Cambridge, MA, USA). The absorbance was measured at 405 nm using a microplate reader. The expression of caspase-3 affected by *X. spinosum* treatment was calculated compared to the result of the negative control [37].

### 4.7. Acute Toxicity of X. spinosum Extract and Fractions

A limit test was carried out to detect the suitable starting doses in the LD₅₀ estimation assay. Briefly, a group (*n* = 2) of female mice (age of six weeks and weight around 20–25 g) was treated with *X. spinosum* extract and fractions (IP injection), with 24 h observation for mortality incidence. If the mice have tolerated the dose, it will increase by 1.5; otherwise, it will reduce by 0.7. The maximum non-lethal and minimum lethal doses presented the lower and upper limits, which were used to prepare LD₅₀ doses [36]. Regarding the LD₅₀ determination assay, three groups (*n* = 6) of mice have injected with IP with various concentrations. For polar extract and fractions (ethanol, aqueous, and aq. Methanol), the doses were 500, 4000, and 7500 mg kg^−1^, while for non-polar fractions (chloroform and *n*-hexane), the doses were 100, 300, and 500 mg kg^−1^. All the estimated doses were within the higher and lower range determined in the limit test. The conditions of mice were monitored for 24 h. The LD₅₀ was detected by the concentration that exhibited 50% mortality by applying the arithmetical method of Karber [38].

### 4.8. Antitumor Activity on Experimental Animals

Breast tumor was induced in two groups (*n* = 7/group) of female Balb/C mice using EMT6/cells in a density of 1 × 10^5^ cells in 0.1 mL. The cells were injected subcutaneously. Following ten days, the developed tumors were measured, and dimensions were recorded. The groups were divided into the control group (*n* = 7) with no treatment and the five treatment groups (*n* = 7) with *X. spinosum* extract and fractions. The estimated dose of ethanol extract represented 10% of the calculated LD₅₀. The treatment stage continued for ten days, and before sacrificing the mice, tumor measurements were recorded. All tumors were extracted and kept in 10% formalin. The volumes of the tumors were recognized using the following formula (2):

(2) $$\mathrm{Volumes of the tumors}\;=\;A\;\times \;B^{2}\;\times \;0.5$$

whereas (A) = the length of the longest aspect of the tumor, (B) = the length of the perpendicular to A [39].

### 4.9. Assessment of Kidney and Liver Functions in Mice

In order to evaluate nephrotoxicity in treated mice, the serum level of creatinine was determined. On the other hand, alanine transaminase (ALT) and aspartate transaminase (AST) were measured to assess liver function. These biomarkers were quantitatively detected by following the instructions of specific kits using DiaSys Respons 920 analyzer (Holzheim, Germany).

### 4.10. Preparation of Murine Splenocytes

The spleen was extracted from a Balb/C mouse (three Balb/C mice were used in this preparation) and ground under sterilized conditions. Cell suspension of spleen cells was prepared using RPMI-1640 media. After centrifugation, the cells pellet was re-suspended in 5 mL of red blood cells lysis buffer (1 mol L^−1^ NH4Cl) (ChemCruz, Santa Cruz, CA, USA), with gentle pipetting many times. The suspension was centrifuged for 8 min at 2000 rpm and 4 ℃. RPMI-1640 media was used to re-suspend the splenocytes cells. The splenocytes were ready to be counted and seeded for the study assays.

### 4.11. Lymphocytes Proliferation Assay

Evaluation of lymphocyte proliferation was achieved based on an MTT-based assay, following a previously described method [40]. The splenocytes suspension (2 × 10^6^ cells/mL) was seeded into a flat-bottomed 96-well plate. Anyway, 5 μg mL^−1^ of concanavalin (Con A) and 4μg/mL of lipopolysaccharide (LPS) were utilized as mitogens for T and B lymphocytes, respectively. The cells were treated with *X. spinosum* extract and fractions (25–100 mg mL^−1^) by adding 100 μL/well from the treatment solution and incubated for 48 h under controlled conditions (5% CO₂ and 37 ℃). Then, 10 μL of MTT solution was added to each well and incubated for 3 h. The final step was adding 100 μL of DMSO to dissolve the formazan particles. The plate was incubated for 1 h to complete the reaction. Using an ELISA microplate reader at 550 nm, the absorbance was measured. The negative control in this experiment was the cells with no treatment. The calculated results were described as the stimulation index compared to the negative control [41].

### 4.12. Macrophage Isolation from Peritoneal Fluid

**Figure 10:**
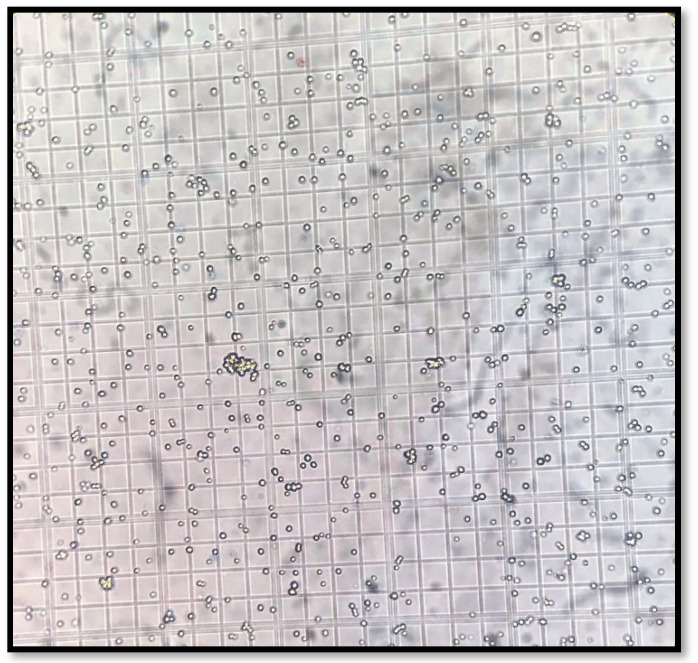
Trypan blue exclusion method shows the viable macrophage cells using a light microscope with 100X power of magnification.

Three days before peritoneal macrophages (PEM) harvesting, Balb/C mice were IP injected with 5 mL of 3% (*w*/*v*) brewer thioglycollate medium (Laboratory Chemicals, Bernd Kraft GmbH, Germany). Ice-cold sterile phosphate–buffer saline (PBS) (PAN-biotech, Aidenbach, Germany) (pH 7.4) was used to isolate peritoneal macrophages by injecting the PBS into the mouse cavity and recollecting the fluid containing the cells. After centrifugation of the collected fluid for 8 min at 2000 rpm and 4 ℃, the formed pellet was re-suspended in a complete RPMI-1640 medium [42]. In this context, the trypan blue exclusion method was performed to have a suitable cell count for the study assays. By using 100X magnification of a light microscope (Nicon, Japan), clear, bright cells were counted while dark blue cells were excluded. Calculations were carried out to count the number of viable cells in the total sample (Figure 10).

### 4.13. In Vitro Phagocytic Assay (Nitro Blue Tetrazolium (NBT) Reduction Test

The nitro blue tetrazolium (NBT) reduction assay was carried out based on Rainard’s research [43]. Briefly, peritoneal macrophages (5 × 10^6^ cells/well) were seeded into a 96-well plate accompanied by various concentrations of *X. spinosum* extract and fractions (25 –100 mg mL^−1^). The plate was incubated for 48 h at 37 °C. Then each well was treated with 20 μL yeast suspension (5 × 10⁶ cells/well in PBS) and 20 μL nitro blue tetrazolium (NBT) (1.5 mg mL^−1^ in PBS), except the negative control wells, which received 20 µL PBS and 20 µL DMSO. After incubation for 1 h at 37 ℃, the supernatant was discarded, and the attached macrophages were washed with RPMI 1640. The cells were air-dried before 120 μL of 2M KOH, and 140 μL DMSO was added to each well. The absorbance was measured at 570 nm using an ELISA microplate reader. The percentage of NBT reduction (phagocytic activity) was estimated according to the following Equation (3) [41]:

Phagocytic index = (OD sample − OD control)/OD control × 100(3)

### 4.14. Determination of Pinocytic Activity Using the Neutral Red Method

Peritoneal murine macrophages were seeded into a flat-bottomed 96-well plate. The cells were treated with *X. spinosum* using three concentrations (100, 50, and 25 mg mL^−1^) and then incubated for 48 h at 37 ℃. A neutral red solution (7.5 mg mL^−1^ in PBS) was added (100 μL) to each well. After incubation for two hours, the supernatant was discarded, and the wells were rinsed with PBS many times to get rid of the remaining particles of the neutral red. Followed by adding 100 μL of cell lysis solution (ethanol and 0.01% acetic acid at the ratio of 1:1) to each well and incubated 24 h at room temperature. At 540 nm, the absorbance was measured using an ELISA microplate reader (BioTek, Highland Park, IL, USA). The pinocytic activity was demonstrated as a pinocytic index. The calculation steps are the same as in the phagocytosis section [43].

### 4.15. TH1/TH2 Assay

In order to detect the effect of *X. spinosum* on different cytokines (IFN-γ, IL-2, IL-4, and IL-10), murine splenocytes were cultured with *X. spinosum* extract, and fractions and immune parameters were determined using mouse uncoated TH1/TH2 ELISA kit (Catalog number 88-7711-44) (Thermo Fisher Scientific, Loughborough, UK). Briefly, murine splenocytes were seeded into a 96-well plate at a concentration of 2 × 10^6^ cells/mL accompanied by adding (1 mg mL^−1^) of each *X. spinosum* extract and fractions and left for 48 h incubation under specific conditions (37 ℃ and 5% CO_2_). After the incubation period, the culture supernatant was collected, and the cytokines level was measured based on the technical procedure mentioned in TH1/TH2 ELISA kit. The concentration (pg mL^−1^) of each cytokine was determined according to an estimated standard curve for each one.

### 4.16. Statistical Analysis

Quantitative data were shown as mean ± standard error. The IC₅₀ values obtained with the different concentrations of plant extract were calculated using nonlinear regression in SPSS (Statistical Package for the Social Sciences, Chicago, Illinois version 24). The statistical significance among the groups was determined using SPSS one-way analysis of variance (ANOVA) and student’s t-test. Differences between groups were considered significant when the *p*-value was less than 0.05 (*p* < 0.05).

## 5. Conclusions

In conclusion, the results of this study suggest that *X. spinosum* shows prominent anticancer and immunomodulatory activities. Such observations are due to the presence of biologically functional compounds in *X. spinosum* extract and fractions. Further research remains necessary to explicate the mechanism of action of the compounds present in the plant and to explore their therapeutic potential in relation to their clinical efficacy, and to have a better understanding of the apoptosis induction effect. Moreover, more phytochemical analysis is needed to determine the exact concentration of each compound.

## Figures and Tables

**Table 1:** LC-MS analysis of *X. spinosum* ethanol extract.

NO	Compounds	Formula	RT (Retention Time)	Relative % Among Detected Compounds (Ethanol Extract)
1	3,5-Dimethoxy-4-hydroxyacetophenone	C10H12O4	4.32	0.183
2	Saponarin	C27H30O15	4.98	0.668
3	Aspalathin	C21H24O11	5.27	0.148
4	Genistin	C21H20O10	5.68	0.399
5	Umbelliferone	C9H6O3	6.02	0.973
6	Naringenin-7-O-glucoside	C21H22O10	6.64	0.546
7	HexA-Apigenin (or Galangin or Genistein) (PUT)	C21H18O11	6.76	1.224
8	Apigenin-7-O-glucoside (Apigetrin)	C21H20O10	6.81	0.980
9	HexA-Chrysoeriol (or Kaempferide) (PUT)	C22H20O12	7.08	1.244
10	7-Glu Chrysoeriol (NMR)	C22H22O11	7.14	0.705
11	Luteolin	C15H10O6	8.65	0.195
12	Sorbifolin	C16H12O6	9.07	0.509
13	Apigenin	C15H10O5	9.93	0.696
14	Hispidulin	C16H12O6	10.35	0.506
15	Eupatilin	C18H16O7	12.59	89.824
16	Kumatakenin	C17H14O6	14.1	1.193

**Table 2:** LC-MS analysis of *X. spinosum* chloroform fraction.

NO	Compounds	Formula	RT	Relative % Among Detected Compounds (Chloroform Fraction)
1	Vanillin	C8H8O3	4.1	0.083
2	(4 or 7) Hydroxy-Coumarin Plus Hydrate	C9H6O3	6.01	0.081
3	HexA-Chrysoeriol (or Kaempferide) (PUT)	C22H20O12	7.13	0.047
4	7-Glu Chrysoeriol (NMR)	C22H22O11	7.2	0.050
5	Hydrocinnamic acid	C9H10O2	7.83	0.049
6	naringenin	C15H12O5	9.52	0.157
7	5,6,4′-Trihydroxy-7,3′-dimethoxyflavone	C17H14O7	9.63	17.767
8	Apigenin	C15H10O5	9.89	0.386
9	6,8-Dimethyl-4-hydroxycoumarin	C11H10O3	10.44	0.050
10	6,8-Dimethyl-4-hydroxycoumarin	C11H10O3	11.51	0.040
11	3-Oxocostusic acid	C15H20O3	11.9	0.045
12	Eupatilin	C18H16O7	12.49	76.839
13	Isobavachin	C20H22O4	14.01	0.042
14	Zapotinin	C18H16O6	15.67	3.966
15	Pygenic acid B	C30H48O5	18.48	0.043
16	Glc-Glc-octadecatrienoyl-sn-glycerol (isomer 1) (PUT)	C33H56O14	20.27	0.078
17	Hederagenin	C30H48O4	21.49	0.063
18	Ursolic acid	C30H48O3	27.98	0.132
19	y-Linolenic acid	C18H30O2	28.14	0.074

**Table 3:** LC-MS analysis of *X. spinosum n*-hexane fraction.

NO	Compounds	Formula	RT	Relative % Among Detected Compounds (*n*-Hexane Fraction)
1	Acteoside	C29H36O15	5.93	4.301
2	Baicalein	C15H10O5	9.81	5.670
3	5,6,4′-Trihydroxy-7,3′-dimethoxyflavone	C17H14O7	10.49	4.510
4	Eupatilin	C18H16O7	12.39	85.517

**Table 4:** LC-MS analysis of X. spinosum aq. Methanol fraction.

NO	Compounds	Formula	RT	Relative % Among Detected Compounds (Aq. Methanol Fraction)
1	Succinic acid	C4H6O4	0.96	0.771
2	Chlorogenic acid	C16H18O9	2.02	0.279
3	4-Hydroxybenzoic acid	C7H6O3	2.38	0.331
4	4-Hydroxybenzoic acid	C7H6O3	2.53	0.462
5	Saponarin	C27H30O15	4.56	0.732
6	Apiin	C26H28O14	4.83	31.047
7	Benzoic acid	C7H6O2	5.45	0.271
8	6,7,3′,4′-Tetrahydroxyflavanone	C15H12O6	5.47	1.898
9	3-Hydroxy-4-methoxycinnamic acid (isoferulic acid)	C10H10O4	5.47	3.732
10	Salicylic acid	C7H6O3	5.78	0.331
11	Acteoside	C29H36O15	5.92	48.386
12	Hesperidin	C28H34O15	6.1	0.336
13	Naringenin-7-O-glucoside	C21H22O10	6.51	1.977
14	Naringin	C27H32O14	6.61	0.367
15	3′,4′,7-Trihydroxyisoflavone	C15H10O5	6.78	0.582
16	HexA-Apigenin (or Galangin or Genistein) (PUT)	C21H18O11	6.79	3.525
17	HexA-Chrysoeriol (or Kaempferide) (PUT)	C22H20O12	7.11	4.708
18	Baicalein	C15H10O5	9.82	0.256

**Table 5:** LC-MS analysis of *X. spinosum* aqueous fraction.

NO	Compounds	Formula	RT	Relative % Among Detected Compounds (Aqueous Fraction)
1	2,5-Dihydroxybenzoic acid	C7H6O4	1.7	0.102
2	4-Hydroxybenzoic acid	C7H6O3	2.52	1.174
3	Chlorogenic acid	C16H18O9	2.92	1.662
4	Umbelliferone	C9H6O3	3.93	0.546
5	Apiin	C26H28O14	4.89	39.604
6	Ferulic acid (trans)	C10H10O4	5.11	0.202
7	Benzoic acid	C7H6O2	5.29	0.093
8	Scutellarein-7-glucuronide	C21H18O12	5.86	5.724
9	3-O-Neohesperidoside Kaempferol (NMR)	C27H30O15	6.14	0.092
10	3′,4′,7-Trihydroxyisoflavone	C15H10O5	6.82	0.280
11	HexA-Apigenin (or Galangin or Genistein) (PUT)	C21H18O11	6.82	19.858
12	4-Methylumbelliferone	C10H8O3	7	0.698
13	HexA-Chrysoeriol (or Kaempferide) (PUT)	C22H20O12	7.12	19.034
14	Naringin	C27H32O14	7.28	0.186
15	Luteolin	C15H10O6	8.58	0.124
16	5,6,4′-Trihydroxy-7,3′-dimethoxyflavone	C17H14O7	9.59	6.236
17	Apigenin	C15H10O5	9.88	0.565
18	Eupatilin	C18H16O7	9.88	2.810

**Table 6:** IC₅₀ values of *X. spinosum* extract and fractions against different cell lines.

*X. spinosum* Extract and Fractions	MCF-7 IC₅₀ (mg mL^−1^) ± SEM	T47D IC₅₀ (mg mL^−1^) ± SEM	EMT6/P IC₅₀ (mg mL^−1^) ± SEM	Vero IC₅₀ (mg mL^−1^) ± SEM
Ethanol extract	9.3 ± 0.25	12.82 ± 0.24	2.57 ± 0.24	>100
Chloroform fraction	7.09 ± 2.33	9.94 ± 1.23	7.59 ± 0.11	>100
Aqueous fraction	4.78 ± 0.24	10.83 ± 0.02	11.83 ± 0.02	>100
Aq. Methanol fraction	10.68 ± 0.25	8.22 ± 0.62	8.1 ± 0.85	>100
*n*-hexane fraction	4.29 ± 2.95	6.59 ± 0.5	5.52 ± 1.16	>100

**Table 7:** Acute toxicity assay of *X. spinosum* ethanol extract, aqueous and aq. Methanol fractions in mice.

Groups (*n* = 6)	Dose (mg/kg)	Dose Difference (a)	Ethanol Extract			Aqueous & aq. Methanol Fractions		
			No. of Mortality	Mean Mortality (b)	Probit (a × b)	No. of Mortality	Mean Mortality (b)	Probit (a × b)
1	500	0	0	0	0	0	0	0
2	4000	3500	2	1	3500	1	0.5	1750
3	7500	3500	3	2.5	8750	3	2	7000

Mice (*n* = 6) were injected intraperitoneally and observed for 24 h. *n* = number of mice in each group. Dose difference (a) = higher dose − lower dose. Mean mortality (b) = (mortality in the second concentration + mortality in the first concentration)/2.

**Table 8:** Acute toxicity assay of *X. spinosum* chloroform and *n*-hexane fractions in mice.

Groups (*n* = 6)	Dose (mg/kg)	Dose Difference (a)	Chloroform Fraction			*n*-Hexane Fraction		
			No. of Mortality	Mean Mortality (b)	Probit (a × b)	No. of Mortality	Mean Mortality (b)	Probit (a × b)
1	100	0	0	0	0	0	0	0
2	300	200	1	0.5	100	2	1	200
3	500	200	3	2	400	4	3	600

Mice (*n* = 6) were injected intraperitoneally and observed for 24 h. *n* = number of mice in each group. Dose difference (a) = higher dose − lower dose. Mean mortality (b) = (mortality in the second concentration + mortality in the first concentration)/2.

**Table 9:** Effects of *X. spinosum* extract and fractions on tumor size and cure percentage.

Treatment Groups (*n* = 7)	Initial Tumor Size (mm^3^) ± SEM	Final Tumor Size (mm^3^) ± SEM	% Change in Tumor Size	% Of mice with no Detectable Tumor	Number of Deaths	Average Tumor Weight (g)
Negative control	500.5 ± 8.8	1043.8 ± 11.1	108.5	22.2%	1	0.73
*X. spinosum* ethanol extract	433 ± 7.4	306.44 ± 9.8	−29.2	42.8%%	0	0.23
*X. spinosum* aqueous fraction	473.9 ± 8.1	245.2 ± 12.1	−48.2	28.5%	0	0.54
*X. spinosum* Aq. Methanol fraction	482.90 ± 6.4	202.6 ± 9.2	−58.0	42.8%	0	0.29
*X. spinosum* chloroform fraction	558.6 ± 8.3	248.5 ± 10.9	−55.5	57.1%	0	0.39
*X. spinosum* *n*-hexane fraction	499.2 ± 11.5	236.1 ± 13.9	−52.7	42.8%	0	0.24

*n* = 7, mm^3^: cubic millimeter.

## Data Availability

Data is contained within the article.
